# Editorial: Fungal Biofilms in Infection and Disease

**DOI:** 10.3389/fcimb.2021.753650

**Published:** 2021-08-27

**Authors:** Jeniel E. Nett, Carolina H. Pohl

**Affiliations:** ^1^Department of Medicine, University of Wisconsin-Madison, Madison, WI, United States; ^2^Department of Medical Microbiology and Immunology, University of Wisconsin-Madison, Madison, WI, United States; ^3^Department of Microbiology and Biochemistry, University of the Free State, Bloemfontein, South Africa

**Keywords:** Candida albicans, biofilm, polymicrobial, Pseudomonas aeruginiosa, Staphylococcus aureus, Streptococcus mutans

Biofilm formation on biotic and abiotic surfaces is an important virulence factor of pathogenic filamentous fungi and yeasts and contributes to antifungal resistance as well as resistance to host immune responses and environmental stresses. There is thus a need to find novel drugs that are able to inhibit biofilms. One option explored in this Research Topic was the use of caffeic acid phenethyl ester (CAPE), a natural compound from propolis (de Barros et al.). This compound was found to significantly reduce *Candida albicans* biofilm biomass and viable cell count. In addition, CAPE decreased the fungal load in the haemolymph of infected *Galleria mellonella* larvae and stimulated the expression of antifungal peptide genes. These antifungal and immunomodulatory activities were also seen in a murine model of infection, where CAPE not only decreased the levels of *C. albicans* colonization and tissue damage, but also increased the expression of β-defensin, a host peptide with antifungal activity.

In recent years, the polymicrobial nature of many infections involving fungi have gained attention. These polymicrobial biofilms have unique characteristics which may be synergistic or antagonistic, depending on the specific organisms in the biofilm. The increased awareness of the role of interacting microbes in polymicrobial biofilms were highlighted in four articles in this Research Topic. *In vitro*, the interaction between *C. albicans* and *Pseudomonas aeruginosa* is characterised as antagonistic, although this is not always the case *in vivo*. In a study to better understand how the presence of *P. aeruginosa* influences *C. albicans*, the investigators examined the role of the *C. albicans* histone acetylase, Set3. They found that deletion of *SET3* influenced the early stages of *in vitro* biofilm formation and *Candida-Pseudomonas* interactions, but not the morphology of *C. albicans* in mature biofilms (Fourie et al.). In addition, they show that Set3 plays a significant role in the virulence of *C. albicans* in a *Caenorhabditis elegans* infection model, even in the presence of *P. aeruginosa*, shedding light on the difference in interactions observed *in vitro* and *in vivo*.

Some interactions between *C. albicans* and bacteria are synergistic, such as the one between *C. albicans* and *Streptococcus mutans*. This synergism in polymicrobial plaque-biofilms was investigated in the presence of saliva (Kim et al.). The polymicrobial biofilms matured rapidly and were able to maintain an acidic environment, capable of damaging human enamel, while the *S. mutans* biofilms could not. Two other articles focussed on the synergistic interaction between *C. albicans* and *Staphylococcus aureus*. It is known that *C. albicans* oral infection predisposes the host to systemic infection by *S. aureus*. Two *C. albicans* adhesins, Als1 and Als3, as well as secretion of the *C. albicans* peptide, candidalysin, were found to play important roles, not only in the interaction between these two microbes, but also in the subsequent phagocytosis and dissemination of *S. aureus* (Van Dyck et al.). In other work, the role of soluble factors produced by *C. albicans* and *S. aureus* biofilms (single and dual-species) in tumour development and progression was investigated (Amaya Arbeláez et al.). For head and neck squamous cell carcinoma, alterations in specific oncogenes caused tumour suppressor genes to accumulate and promote cancer progression. Using tumour cell lines, the investigators show how soluble factors produced by single and dual-species biofilms impact mammalian cell survival, cell cycle profile, and the expression of proto-oncogenes, suggesting that these organisms may influence carcinoma progression in patients.

Host-biofilm interactions were also explored in the context of innate immunity (Smolarz et al.). During biofilm formation, *C. albicans* produces an extracellular matrix. The investigators isolated fungal DNA from extracellular matrix and examined the impact on human neutrophils. They found that fungal DNA impacts neutrophil function, including the formation of neutrophil extracellular traps, production of reactive oxygen species, and chemotaxis. The study shows how a component of fungal biofilms may greatly influence host innate immunity.

The articles presented in this Research Topic highlight the varied approaches taken by researchers studying the role of fungal biofilms in infection and disease, including formation, regulation and inhibition of biofilm formation (de Barros et al.; Fourie et al.; Kim et al.), the use of non-mammalian infection models (de Barros et al.; Fourie et al.), interaction with the host immune system (de Barros et al.; Smolarz et al.; Van Dyck et al.) as well as the influence of biofilms on cancer cells (Amaya Arbeláez et al.). In addition, the number of articles dealing with the interaction between *C. albicans* and various bacteria highlights the increasing interest of researchers in the polymicrobial nature of many biofilm infections.

## Author Contributions

The authors contributed equally to this work. CP and JE co-wrote the manuscript. All authors contributed to the article and approved the submitted version.

## Funding

This work was supported by a two National Research Foundation of South Africa grants [grant numbers 118543 and 115566 to CP], the Burroughs Wellcome Fund [1012299 to JN] and the Doris Duke Charitable Foundation [2017074 to JN].

## Conflict of Interest

The authors declare that the research was conducted in the absence of any commercial or financial relationships that could be construed as a potential conflict of interest.

## Publisher’s Note

All claims expressed in this article are solely those of the authors and do not necessarily represent those of their affiliated organizations, or those of the publisher, the editors and the reviewers. Any product that may be evaluated in this article, or claim that may be made by its manufacturer, is not guaranteed or endorsed by the publisher.

